# Supplementary material for: Vitamin D and Calcium Supplementation in Nursing Homes—A Quality Improvement Study
Source: Nutrients. 2022 Dec 16;14(24):5360. doi: 10.3390/nu14245360 (PMC9780874; doi:10.3390/nu14245360)
Supplement: Supplementary file 1 [file nutrients-14-05360-s001.zip › nutrients-2044854-SI.pdf]

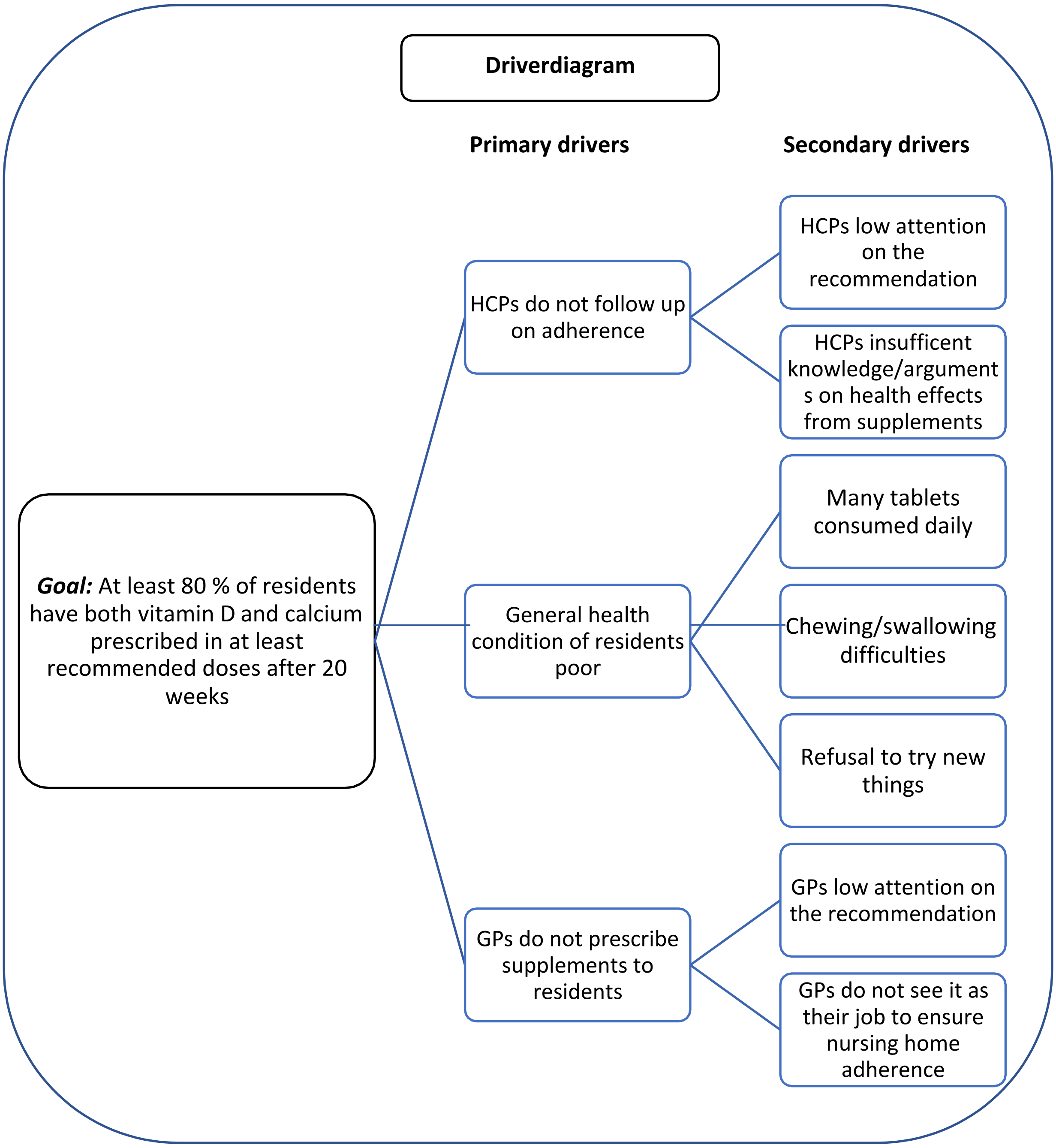

Supplementary Figure S1. Driver diagram including the goal of the project and primary and secondary drivers affecting the goal. *Abbreviations:* GPs, General practitioners, HCPs, Health care professionals.
